# Male Mosquitoes as Vehicles for Insecticide

**DOI:** 10.1371/journal.pntd.0003406

**Published:** 2015-01-15

**Authors:** James W. Mains, Corey L. Brelsfoard, Stephen L. Dobson

**Affiliations:** MosquitoMate, Inc., Lexington, Kentucky, United States of America; Centers for Disease Control and Prevention, Puerto Rico, UNITED STATES

## Abstract

**Background:**

The auto-dissemination approach has been shown effective at treating cryptic refugia that remain unaffected by existing mosquito control methods. This approach relies on adult mosquito behavior to spread larvicide to breeding sites at levels that are lethal to immature mosquitoes. Prior studies demonstrate that ‘dissemination stations,’ deployed in mosquito-infested areas, can contaminate adult mosquitoes, which subsequently deliver the larvicide to breeding sites. In some situations, however, preventative measures are needed, e.g., to mitigate seasonal population increases. Here we examine a novel approach that combines elements of autocidal and auto-dissemination strategies by releasing artificially reared, male mosquitoes that are contaminated with an insecticide.

**Methodology:**

Laboratory and field experiments examine for model-predicted impacts of pyriproxyfen (PPF) directly applied to adult male *Aedes albopictus*, including (1) the ability of PPF-treated males to cross-contaminate females and to (2) deliver PPF to breeding sites.

**Principal Findings:**

Similar survivorship was observed in comparisons of PPF-treated and untreated males. Males contaminated both female adults and oviposition containers in field cage tests, at levels that eliminated immature survivorship. Field trials demonstrate an ability of PPF-treated males to transmit lethal doses to introduced oviposition containers, both in the presence and absence of indigenous females. A decline in the *Ae. albopictus* population was observed following the introduction of PPF-treated males, which was not observed in two untreated field sites.

**Conclusions/Significance:**

The results demonstrate that, in cage and open field trials, adult male *Ae. albopictus* can tolerate PPF and contaminate, either directly or indirectly, adult females and immature breeding sites. The results support additional development of the proposed approach, in which male mosquitoes act as vehicles for insecticide delivery, including exploration of the approach with additional medically important mosquito species. The novelty and importance of this approach is an ability to safely achieve auto-dissemination at levels of intensity that may not be possible with an auto-dissemination approach that is based on indigenous females. Specifically, artificially-reared males can be released and sustained at any density required, so that the potential for impact is limited only by the practical logistics of mosquito rearing and release, rather than natural population densities and the self-limiting impact of an intervention upon them.

## Author Summary

Approximately half of the human population is at risk of dengue. Additional mosquito borne pathogens, *e*.*g*., chikungunya, are spreading globally, as are important mosquito vectors. In the absence of approved vaccines, therapeutant or prophylaxis, vector control remains the only means to combat multiple mosquito-borne pathogens. Auto-dissemination strategies have attracted attention as a method to reduce mosquito populations and benefit from mosquito behavior, in which a female mosquito visits multiple breeding sites. As practiced currently, ‘dissemination stations’ are attractive to adult females, which enter the station, become contaminated with a juvenile hormone analogue (JHA), exit and then contaminate breeding sites with levels of JHA that are lethal to immature mosquitoes. The auto-dissemination method is particularly attractive for those species that breed within small, cryptic sites, which serve as refugia from existing insecticidal measures. Here we examine mathematically and empirically, a novel approach that is not station-based, but which integrates elements of autocidal control. Specifically, the approach would release JHA-contaminated adult male mosquitoes, which do not bite or transmit pathogens. The males deliver JHA to breeding sites, either directly or indirectly, *i*.*e*., via the cross-contamination of females, which subsequently transfer JHA to breeding sites. The examined autocidal method can be used preemptively, *e*.*g*., in areas with low densities of indigenous mosquitoes and in advance of the natural population increase. Unlike auto-dissemination approaches that rely upon the indigenous population, an approach based on artificially-reared males can made more intensive, because the number of males released is limited only by the logistics of male rearing and release and methods for mass-production of mosquitoes are developed already.

## Introduction

Mosquito control remains the only means available to combat some medically important, vector-borne pathogens, such as West Nile, Dengue and Chikungunya viruses, because no approved vaccine, therapeutant or prophylaxis exist [1,2]. Chemical insecticides are used most commonly in mosquito control, with formulations that include larvicides and adulticides (*e*.*g*., space sprays, residual indoor applications, and insecticide-treated bed nets) [3]. With each of these approaches, however, their efficacy can be reduced by an inability to achieve adequate coverage that is needed to effectively reduce pathogen transmission [4–6].

Larvicides are applied to aquatic habitats of developing immature mosquitoes (‘breeding sites’) and are demonstrated to reduce mosquito-borne disease transmission [7–9]. However, in some contexts, its implementation at a programmatic level can be difficult [10]. Specifically, aquatic habitats include a variety of types, many of which can be small, sheltered and difficult to locate and treat (‘cryptic breeding sites’) [11]. Because financial and human resources are limited, it can be difficult to achieve a coverage level sufficient to reduce disease [5,12]. Area-wide broadcasting of larvicides can be used to improve coverage, via aircraft and vehicle-mounted sprayers, but in some situations, broadcasting is constrained by environmental regulations or restrictions, community concerns and mosquito resistance to the active ingredients of existing larvicides [13–16]. Furthermore, some larvicide formulations are expensive, and the large quantities required for broadcasting strategies can be cost prohibitive.

Auto-dissemination has attracted attention due to its potential to address important gaps with existing mosquito control methods. Auto-dissemination is a method of pesticide ‘self-delivery,’ which is premised upon the use of insects as the delivery agent. Insects carrying small amounts of insecticide can deliver an active ingredient to cryptic refugia, rather than human applicators, and this method can require less pesticide relative to broadcasting. For this reason, auto-dissemination approaches have become an important pesticidal method for termites, beetles, and moths [17].

**Figure 1:**
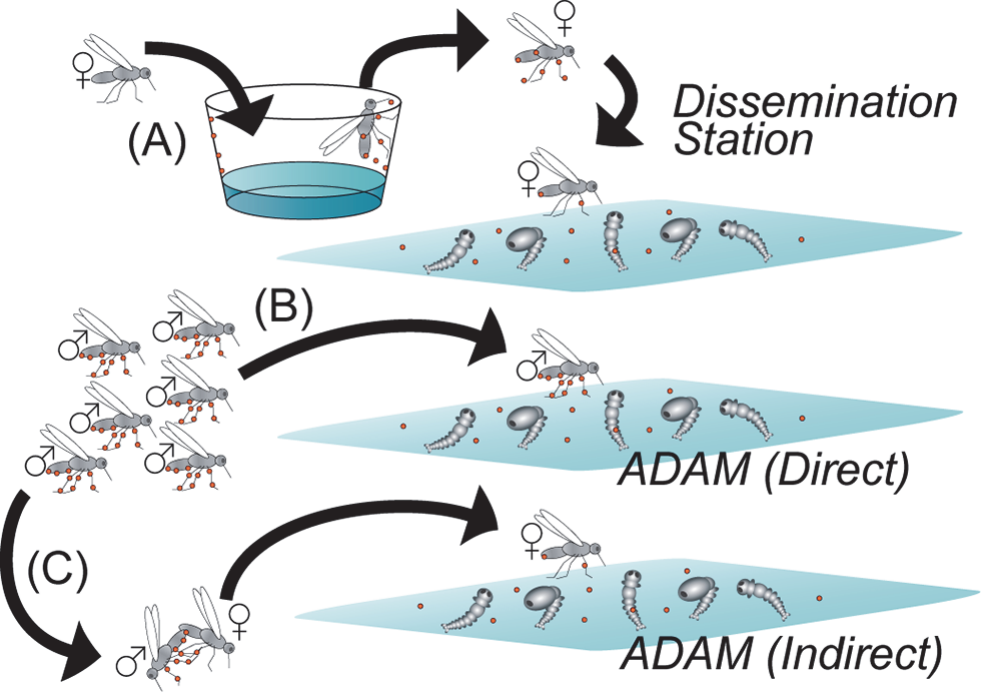
Diagram comparing the auto-dissemination station-based approach with the ADAM approach. In (A), an auto-dissemination station is attractive to indigenous female mosquitoes (grey), which enter and become contaminated with a persistent juvenile hormone analogue (PPF) that does not harm the adult. The PPF-contaminated females (black) exit the trap and transfer the PPF to immature mosquito breeding sites. In (B and C), the ADAM approach is based on manufacturing adult male mosquitoes that are dusted with PPF (black), which are released into a treatment area. The PPF-treated males can then (B) directly transfer PPF to immature mosquito breeding sites and (C) indirectly transfer PPF by cross-contaminating indigenous females, which subsequently transfer the PPF to breeding sites.

Auto-dissemination approaches are being explored for mosquitoes [18,19]. Proposed auto-dissemination methods are based on the behavior of adult mosquitoes and their attraction to breeding sites, including cryptic sites that human operators may often fail to find. As currently practiced, auto-dissemination consists of placing artificial adult resting sites (‘dissemination stations’) that are (1) attractive to adult mosquitoes and (2) are treated with a persistent juvenile hormone analogue (JHA) [20]. Upon entering the dissemination station (Fig. 1A), the adult mosquitoes become contaminated with the JHA, which is not acutely toxic to the adult. The JHA is lethal to immature mosquitoes, when their breeding sites become contaminated by the females that arrive to lay eggs and introduce the JHA. An additional approach is based on treated bed nets [21].

Models predict a multiplicative ability of the auto-dissemination approach to achieve high breeding site coverage, despite covering a relatively small proportion of the resting sites. The coverage of breeding sites (*C*_h_) is related to the coverage of adult resting sites (*C*_r_), the duration for which habitats remain unproductive after contamination (*U*), the number of ovipositions by the mosquito population (*O*) relative to the number of habitats (*H*), and the mean number of contaminated ovipositions required to render a breeding site unproductive (Ω) [19,22].

$$C_{h}=1-e^{-C_{r}UO/H\Omega }$$ (1)

This relationship shows that a majority of breeding sites can be affected, even when treating a minority of resting sites, if the pesticide is durable (*U* ≥ 7 days), the mosquito abundance and habitat availability is such that breeding sites are contaminated more than once per day (*O/H* ≥ 2) and one contamination is sufficient to render a habitat unproductive (Ω = 1).

In addition to modeling, field trials by multiple research groups have demonstrated the efficiency of the auto-dissemination approach, showing that (1) mosquitoes become contaminated using different dissemination station designs and (2) that the contaminated mosquitoes can transmit lethal doses of the pesticide [19,20]. Importantly, prior studies demonstrate also that (3) adult male mosquitoes are attracted to and are contaminated by auto-dissemination stations, (4) that males can venereally transfer JHA to females upon mating and (5) that the venereally-contaminated females can subsequently transfer lethal concentrations of JHA to breeding sites [18].

The most commonly used JHA in the auto-dissemination approach is pyriproxyfen (PPF), which does not affect contaminated adults, *i*.*e*., it is neither lethal nor repellant [23]. In contrast to adults, the concentration required to prevent mosquito development (LC_50_) is 0.012 parts per billion [23]. At this rate, 32mg of PPF would be adequate to treat an olympic-size swimming pool (2.5 million liters). And 1/1,000th of the dry weight of a mosquito adult would be adequate to treat a 200ml breeding site [24]. The residual activity of PPF is four months in water [25]. Little resistance to this chemical class has been observed in mosquitoes [26,27]. PPF is relatively safe for non-targeted organisms, including vertebrates [28]. The World Health Organization has approved PPF for use in drinking water [29].

Here, we use models to consider the limitations of current station-based auto-dissemination approaches and to propose an additional auto-dissemination method that is based upon the release of PPF-treated male mosquitoes (Fig. 1). Inundative male releases are feasible, because in mosquitoes, the males do not bite or transmit pathogens to the human population [30]. The approach of releasing PPF-treated male mosquitoes is subsequently referred to as “Auto-Dissemination Augmented by Males” (ADAM). We examine empirically (1) the effects of PPF on the survival of male *Aedes albopictus* which serve to contaminate breeding sites, (2) the ability of treated males to directly contaminate larval breeding sites, *i*.*e*., even without females and (3) the ability of treated males to transfer PPF to females, at dosages adequate to lethally contaminate larval breeding sites. The results encourage additional examination and development of this approach as an additional tool against important mosquitoes.

## Methods

*Ae*. *albopictus* mosquitoes used in experiments were from a colony established in Lexington, Kentucky in 2011 and named the “Wildcat” (WC) strain. Larvae were fed with a 60g/L liver powder (ICN Biomedicals, CA, USA) suspension *ad libitum*. Adults were held in 24.5 cm^3^ cages (MegaView Science Co., Taichung, Taiwan) and provided a constant supply of 10% sucrose. Adult mosquitoes used in experiments were between one and two days post emergence. The PPF treatment consisted of a 30:70% mixture of Esteem 35 WP IGR (Valent Biosciences, IL, USA) and DayGlo (Dayglo Color Corp., Cleveland, OH), respectively. The PPF was applied to mosquitoes housed in a 1 L enclosed cardboard container using a PowerPuff insufflator for approximately 5 sec (Gremar Power Puff 898, Gremar Inc., W. Des Moines, IA). Dayglo is routinely used for mosquito marking (*e*.*g*., mark release recapture experiments) [31,32] and facilitates the subsequent tracking of the dust mix. We note that PPF is a pupacide. However, PPF is registered and commonly referred to as a ‘larvicide,’ and therefore we use the latter terminology here.

For longevity studies, adult male mosquitoes were dusted with PPF (treatment) or left undusted (control). Three replicate control and treatment groups of 15 males/each were put into cages with a 10% sucrose solution and monitored for adult survivorship.

For field cage trials, ten oviposition cups were placed within each field cage (10’x12’; Ozark Trail, CA, USA), along with a 10% sucrose solution. Five oviposition cups/cage were covered with bridal veil to exclude mosquito entry. The remaining ovisites were identical, but uncovered. Ovisites consisted of black 0.5 L plastic cups (Solo Cup Co., USA) lined with germination paper (Anchor Paper Co., USA) and with 250 ml water.

For the field cage experiments, young adult male mosquitoes (≤2d post eclosion) were treated with the Esteem/DayGlo mixture, as in the longevity study, and then introduced into cages (50 males/cage). Twenty-four hours after male release, 50 newly-eclosed virgin females were added to the field cages. Five days after establishment, all adults were evacuated from the field cages using a modified aspirator [33]. Adults were killed by freezing and observed for dust. Oviposition cups and associated egg papers also were examined for dust.

To test for the presence of larvicidal activity, bioassays were performed in 20 ml scintillation vials (#986540; Wheaton Millville, NJ) with 15 ml water, 0.2 ml liver powder solution and four second instar WC larvae. For bioassays of the adults that were removed from field cages, the removed adults were killed by freezing and then placed individually into bioassay vials, and assays were observed for immature survivorship. For bioassays of ovisites, each cup was separated into three components: water, germination paper lining and cup. For bioassays of ovisite water, two replicate 15 ml water samples were drawn from each ovisite and combined with 0.2 ml liver powder solution and four second instar WC larvae in scintillation vials, and immature survivorship was monitored. To examine for PPF on germination papers, each paper was removed from the ovisite and then submerged in 200 ml water with four second instar WC larvae and 1 ml liver powder solution, and immature survival was monitored. With the water and germination papers removed, each ovisite was examined for PPF by rinsing with 200 ml water, and 15 ml of the resulting rinsate was combined with 0.2 ml liver powder solution and four second instar WC larvae in scintillation vials, and immature survivorship was monitored.

Negative control assays consisted of an undusted male adult added to 15 ml water, 0.2 ml liver powder solution and four second instar WC larvae in a scintillation vial. Positive control assays were the same as negative control assays, but with the addition of a single male, freshly dusted with PPF powder.

Field studies were conducted at two sites in Lexington, KY. The adult population was monitored via weekly 24 hour collections using BG traps (Biogent Sentinel, Regensburg, Germany). Artificial ovisites were as described for field cages. Water samples were removed weekly from ovisites and bioassayed as described above. In the second field study, 10-minute landing collections were conducted to observe for *Ae*. *albopictus* females. WC males introduced at field sites were treated with the Esteem/DayGlo mixture, as described in the longevity study.

Statistical analyses were performed using JMP 9.0.1 and SAS 5.1 software (SAS Institute, Cary, NC). Kaplan-Meier, Log-Rank was used for analysis of adult longevity. Non-parametric analysis was used (Kruskal-Wallis and Wilcoxon) to examine results of bioassays. To assess population trends following treatment, a repeated measures ANOVA was conducted, followed by a linear regression of the female adult number [LN(females+1)] by Collection Week. To examine for an effect of proximity to release site, a linear regression was made between bioassay lethality and distance from the release point.

## Results

### Mathematical Consideration

As currently practiced against mosquito populations, models predict that station-based auto-dissemination relies upon a vigorous, naturally-occurring mosquito population. Specifically, indigenous mosquitoes must enter the station, become contaminated with the larvicide and then deliver the larvicide to breeding sites. Mosquitoes are the vehicle for the larvicide; therefore, in areas with lower mosquito densities, the larvicide may not be effectively delivered to breeding sites. Using the model above (Equation 1), the efficacy against the mosquito population is directly related to the number of ovipositions per population (*O*) [34]. Therefore, the model predicts that the impact of this approach to affect potential breeding sites will be limited, until there are adequate adults to become contaminated and transmit the pesticide (S1 Fig.). If these predictions are correct, this can limit station-based approaches as a preventative control tool. Prior laboratory work examining the relationship between adult mosquito number and accumulation of pesticide support model predictions [19].

The model predicts also that a station-based approach based solely upon naturally-occurring mosquitoes can be a victim of its own success. This is evident from the endogeneity within Equation 1, in which the coverage term on the left side of the equation is interdependent with the mosquito density term on the right side of the equation. Specifically, when introduced into areas of high mosquito activity, a successful station-based auto-dissemination approach that reduces the mosquito population will reduce the number of ovipositions by the mosquito population (*O*). Assuming that the number of potential breeding sites (*H*) remains constant within the habitat, fewer females will result in fewer ovipositions, which is predicted to reduce the coverage of breeding sites (S1 Fig.). Efficacy differences between field sites observed in prior field trials of a station-based approach were suggested that differing abundance of mosquitoes between the sites as a possible explanation [19,20].

The model predicts that a method offsetting the above predicted limitations would be to artificially sustain mosquito activity through the release of mosquitoes. By maintaining high mosquito activity, the delivery of the larvicidal agent can continue. Clearly however, sustaining a population through the release of female mosquitoes, which bite and could transmit pathogens, would be an unacceptable approach. However, the introduction of adult male mosquitoes, which do not bite or transmit pathogens, is feasible. There are multiple vector-control strategies that are based on the repeated, inundative release of adult male mosquitoes, including Sterile Insect Technique (SIT) and newer strategies based on transgenic mosquitoes (*e*.*g*., RIDL) [35].

Furthermore, because the males are reared artificially and released, the ADAM approach would provide opportunity for their direct treatment with the larvicide, prior to their release, rather than relying on dissemination stations to contaminate males. Direct treatment in a controlled environment can permit a more uniform and standardized application of the larvicide, relative to a station-based approach.

### Empirical Examination of the ADAM Approach

**Figure 2:**
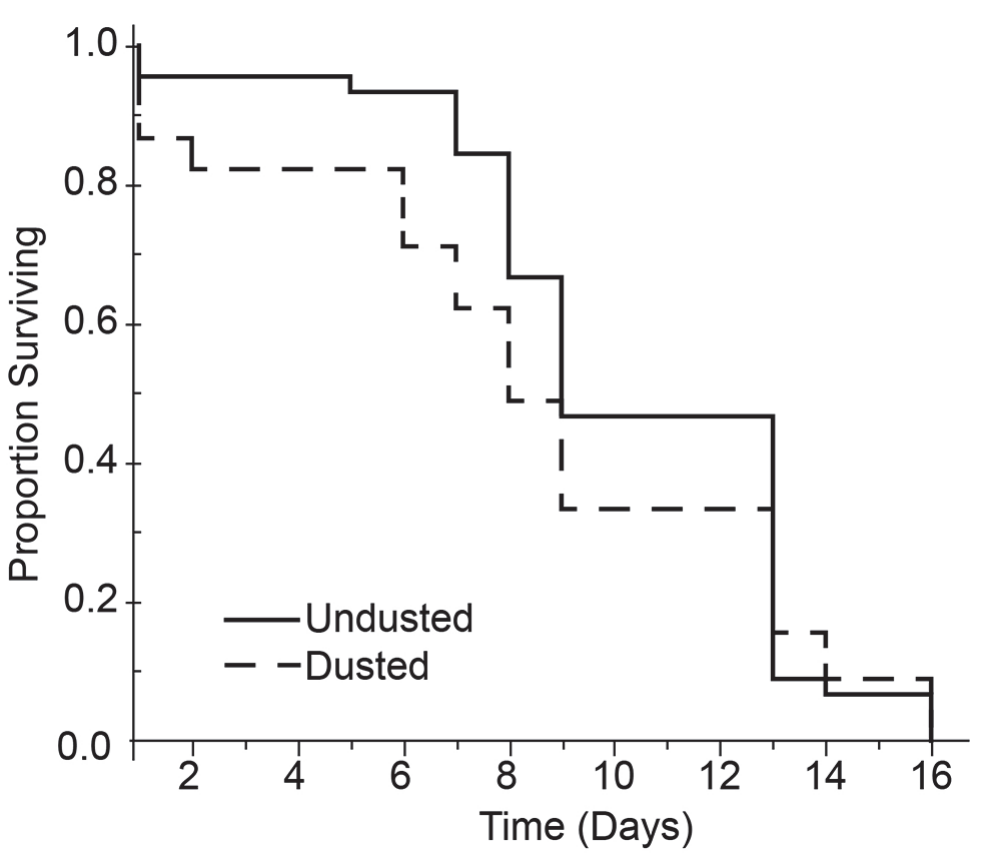
Survivorship of male *Ae*. *albopictus* treated with Pyriproxyfen/Dayglo dust compared to untreated males.

**Effect of PPF dust on adult male survival.** Critical to the proposed ADAM approach, the laboratory-reared male adults must remain competitive after treatment with the insecticide. To examine for an acute effect on survival, three replicate groups of males were divided and either dusted with PPF (treatment) or not dusted (control). Following treatment, mosquitoes were held in the laboratory and observed for male mortality. As illustrated in Fig. 2, comparing treatment and control groups, male survivorship was not observed to differ (Kaplan-Meier, Log-Rank, *p* > 0.32).

**Transfer of PPF from males to females and ovisites.** In an additional experiment, field cages were used to examine the ability of PPF-treated males to interact with and cross contaminate females. PPF-treated males were introduced into field cages. Untreated females, *i*.*e*., not PPF-treated, were introduced into cages also. Field cages included ten oviposition sites (‘ovisites’), five of which were covered with screen to prevent mosquito entry.

**Figure 3:**
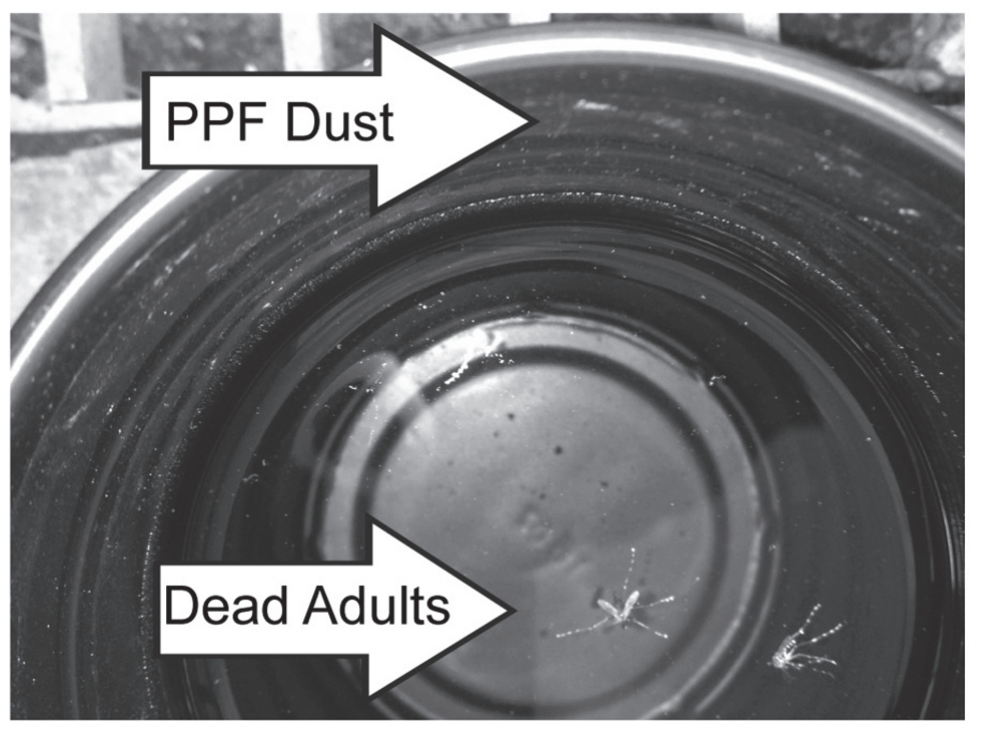
An example of an ovisite removed from a field cage trial. Arrows point to pyriproxyfen dust on the sides of the cup and to two dead adult *Ae*. *albopictus* floating in the water.

Five days after mosquito introduction into cages, adults and ovisites were removed from the cages. Ovisites were visually examined for PPF powder residue (Fig. 3). No PPF powder was observed in the screen-covered ovisites, *i*.*e*., from which mosquitoes were excluded (n = 15 cups). In contrast, PPF powder was observed in all but one of the unscreened cups, *i*.*e*., in one cage replicate, no powder was observed in one of the five unscreened cups. Dead adults (n = 5) were observed in two of the unscreened cups (Fig. 3) and none of the screened cups.

**Figure 4:**
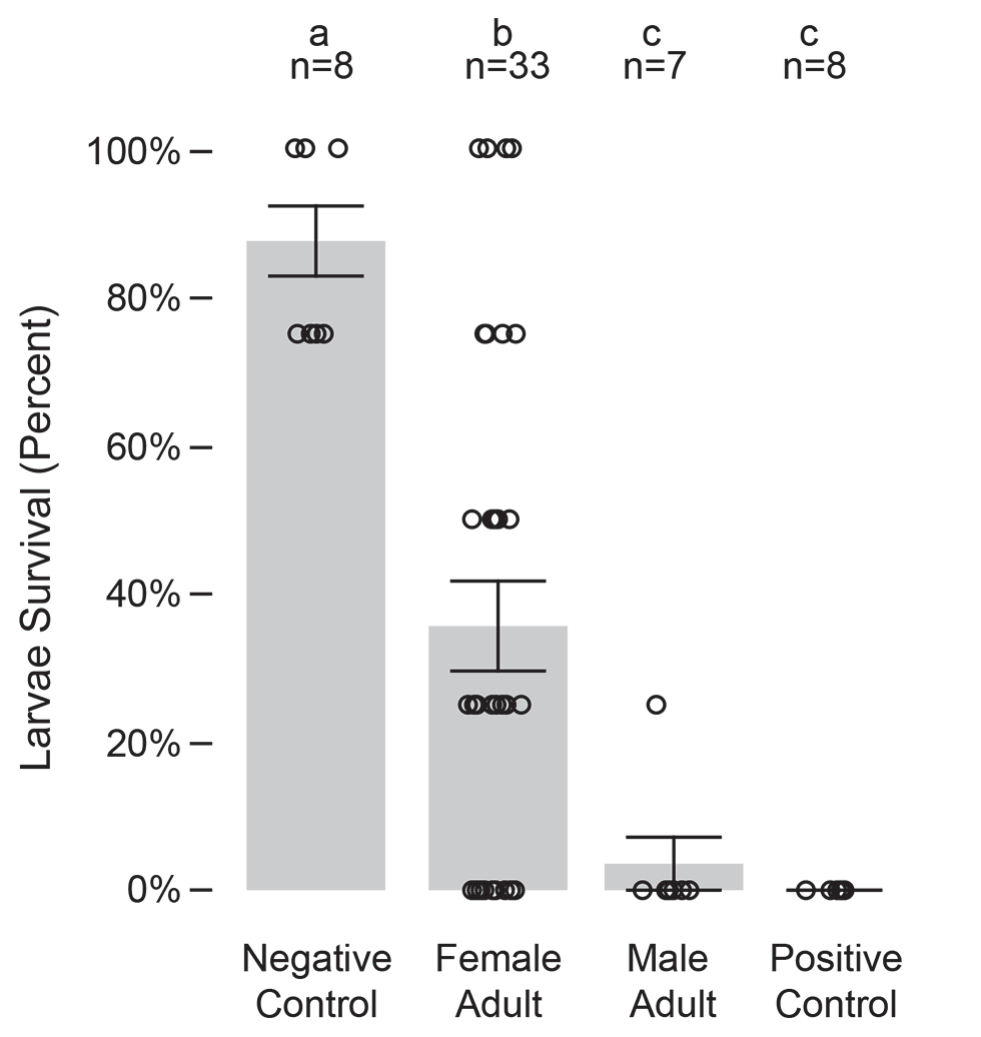
Adults removed from field cages were examined for insecticidal activity using immature bioassays. Letters above the bars indicate significant differences (*p* < 0.01, Wilcoxon). The number of replicates is shown above each column. Bars show standard errors.

Adults removed from cages were bioassayed for toxicity against *A*. *albopictus* larvae. The bioassay results demonstrate significantly higher mortality of larvae exposed to males and females removed from field cages, relative to the negative control bioassays (*p* < 0.0001, Kruskal-Wallis). The survivorship of larvae exposed to adult females from field cages was 35.6±6.1%, mean±std err (Fig. 4). In contrast, higher survival of immatures was observed in bioassays not receiving an adult from field cages, *i*.*e*., negative control, was 87.5±4.7%. Adult males removed from cages and introduced into immature bioassays resulted in 3.6±3.6% immature survivorship, which was not significantly different from the positive control assays (0±0% immature survivorship). No difference was observed between the three field cage replicates (*p* > 0.2, Kruskal-Wallis).

In addition to the living adults removed from field cages, five dead males were recovered from unscreened cups, floating in the water. Bioassays with the latter five males were 100% lethal to exposed larvae in bioassays, with no larvae surviving in bioassays (n = 5).

**Figure 5:**
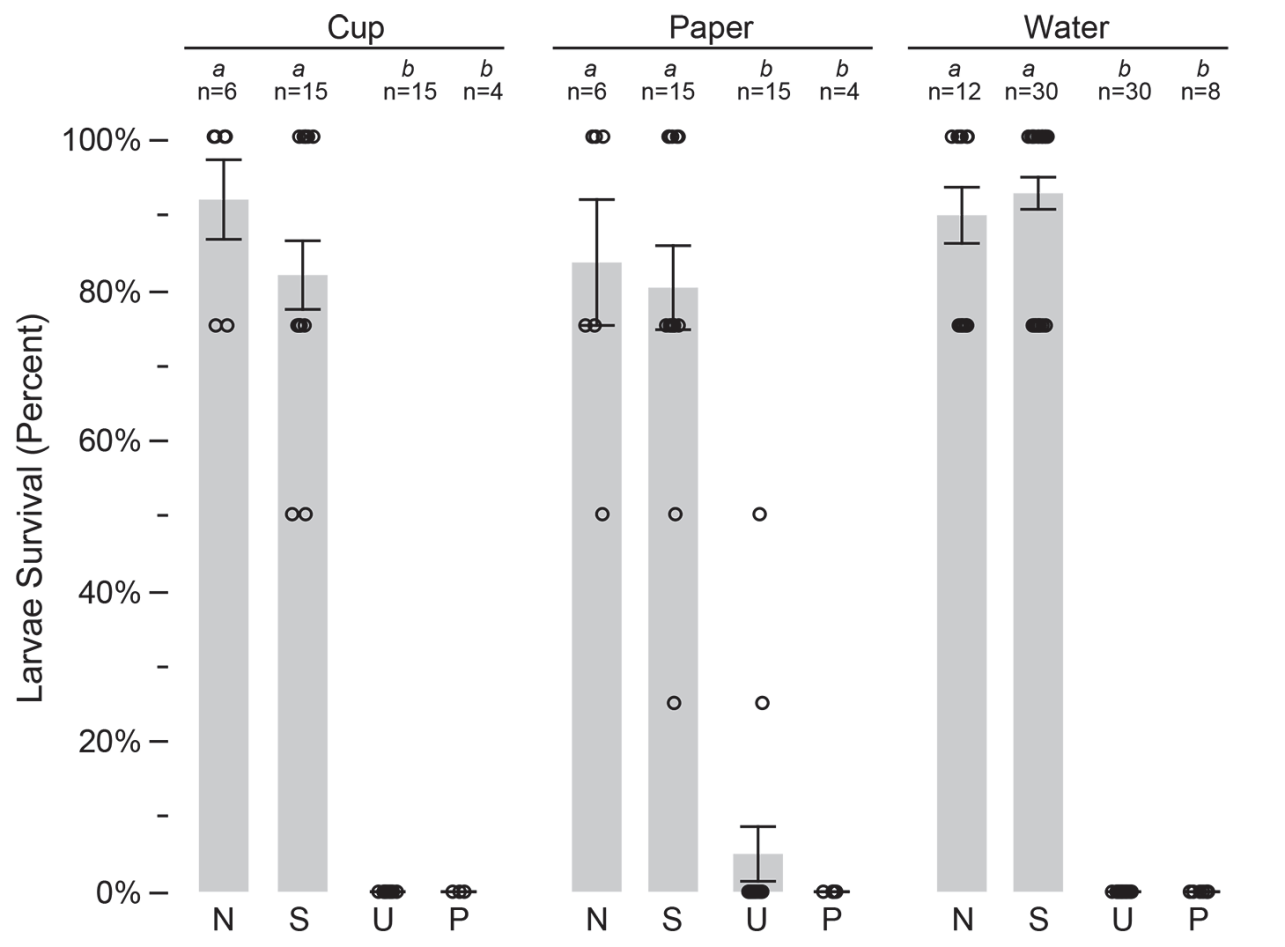
Oviposition cups removed from field cages were separate into three different components (*i*.*e*. cup, paper lining, and water), and each was examined for insecticidal activity using immature bioassays. For each component, screened (S) and unscreened (U) cups are compared, along with the negative (N) and positive (P) controls. Letters above the bars indicate significant differences (*p* < 0.01). The number of replicates is shown above each column. Bars show standard errors.

As described above, PPF powder residue was observed in the majority of the unscreened ovisites at the end of the experiment. Therefore bioassays were conducted to examine for PPF residue introduced into the ovisites. For assays, each cup was separated into three components: water, paper lining and cup. Water samples removed from cups were tested by bioassay, with two samples per cup. As shown in Fig. 5, high immature survivorship (92.5±2.1%) was observed in bioassays of water from screened cups, *i*.*e*., mosquitoes excluded. In contrast, no larvae survived in assays of water samples removed from unscreened cups. Comparison with the control groups show no difference between the unscreened and positive control (Fig. 5). The results from screened cups did not differ significantly from that of the negative control bioassays. Similar to the results of the water bioassays, assays of the germination paper lining and the surface of the unscreened cups were highly lethal to larvae in bioassays, not different from that observed in the positive control group (Fig. 5). In contrast, high survivorship was observed in assays of the screened cups, similar to that of the negative control group.

**Figure 6:**
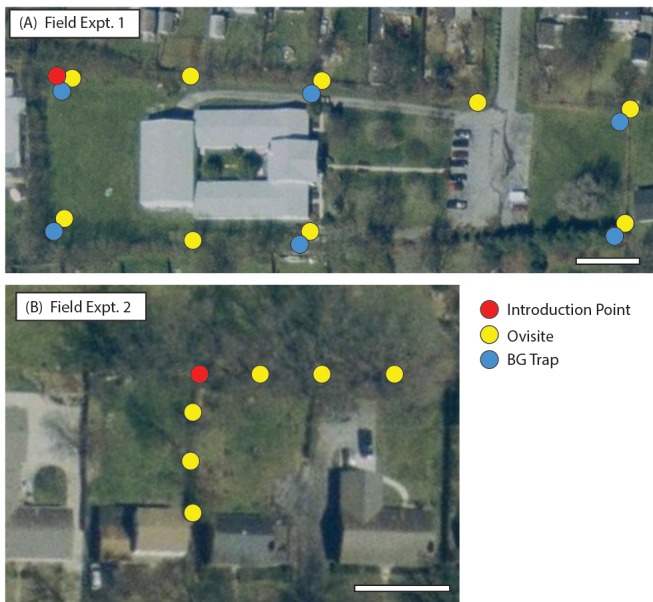
Field sites for male introduction experiments in Lexington, Kentucky. (A) Field Experiment 1 Site consisted of a single point introduction site (red circle), six BG trap sites (blue circle) and nine ovisites (yellow circles). (B) Field Experiment 2 Site consisted of a single point introduction site and six ovisites. Images are from http://datagateway.nrcs.usda.gov/GDGHome.aspx. Bars = 60ft.

**Figure 7:**
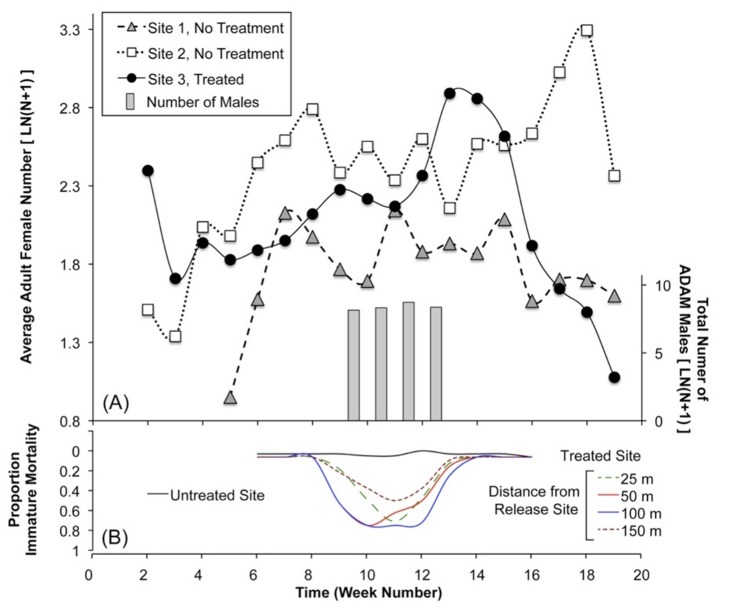
Results of Field Trial 1. (A) *Ae*. *albopictus* adults are monitored using BG traps at three sites. Grey bars indicate introductions of PPF-dusted males at Site 3 only. Beginning after Week 13, a consistent population decline is observed at Site 3, which is not observed at the untreated sites. Lines show a three-week moving average for the adult collections. (B) Bioassays of artificial oviposition sites show increased larval mortality up to 150m from the Site 3 release point. In contrast, low mortality is observed in bioassays of ovisites within untreated areas.

**Field trial of the ADAM approach.** Based on field cage results, open releases of PPF-males were conducted, to examine the ability of males as PPF carriers in the field. At the treatment site (Fig. 6A), ovisites and BG traps were positioned at variable distances from a release point. Additional ovisites were monitored at two untreated sites >4 km from the treatment site. Following nine weeks of pre-introduction population monitoring, an average of 4,500 PPF-treated males/week were introduced at the Treatment site (Fig. 7A). An immediate toxic effect was observed in water sampled from ovisites from the Treatment site (Fig. 7B), while bioassays of water from the Untreated site continued to result in good survival (>80%; Fig 7B). Toxicity at the Treatment site persisted for the duration of the release period.

No immediate impact on the adult population was observed following the introduction of PPF-dusted males. However, a decline in the adult population at the Treatment site was observed (Fig. 7A), beginning approximately four weeks after the initial ADAM male introduction, *i*.*e*., beginning at Week 14. In contrast, a decline was not observed in the populations at the Untreated sites; instead these populations continued at densities similar to that observed during the pre-introduction period. Repeated Measures ANOVA shows no significant SITE effect, but there were significant effects by WEEK, F_(6,36)_ = 5.8; *p* < 0.0002 and the WEEK*SITE interaction, F_(6,36)_ = 6.0; *p* < 0.0002. Linear regression of the two Untreated sites during Weeks 14–20 were either non-significant or significantly positive, *i*.*e*., increasing population (S2 Fig.). At the Treatment site, however, the regression during the same period was significantly negative following male introduction (S2 Fig.). At Week 21, the ambient air temperature dropped to 6°C, and no additional mosquitoes were collected at any of the sites.

**Examining adult males as direct carriers.** An important characteristic of the ADAM approach would be the ability of males to deliver insecticide directly to breeding sites, in the absence of females. In the preceding study, because both females and males were present, the PPF contamination could have resulted from indirect contamination by males, *i*.*e*., males cross-contaminate females, and the females subsequently contaminate breeding sites. Therefore, to examine the ability of males to act as ‘direct carriers,’ delivering PPF directly to breeding sites, a second field study was performed in early spring, before the indigenous population was observed.

Similar to the prior field experiment, ovisites were placed at varying distances away from a release point (Fig. 6B), and water was sampled weekly and assessed in larval bioassays. An additional array of ovisites was deployed and tested at an Untreated site, which was >4 km from the Treatment site. Water samples were drawn for two weeks prior to male introduction and two weeks during male introduction. To monitor for the appearance of the indigenous population, landing counts were performed weekly at both the Treated and Untreated sites.

**Figure 8:**
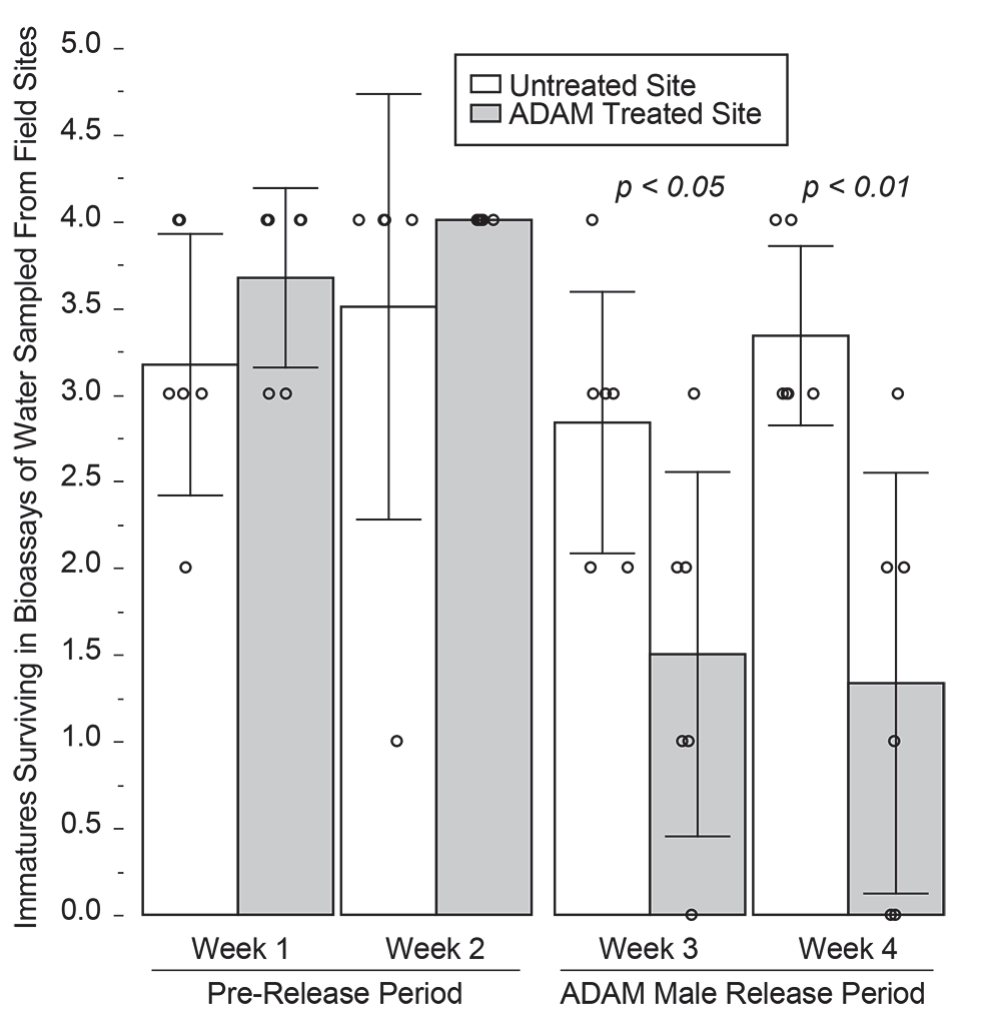
In the absence of an indigenous *Ae*. *albopictus* population, *i*.*e*., Field Trial 2, increased immature mortality is observed following the introduction of PPF-treated males. During the pre-release period, good larval survival is observed in bioassays of water sampled from ovisites at both the Untreated and Treated sites. Following male introductions, reduced survival is observed at the Treated site only. Significant differences are indicated above the columns (Wilcoxon). Bars show standard deviation.

**Figure 9:**
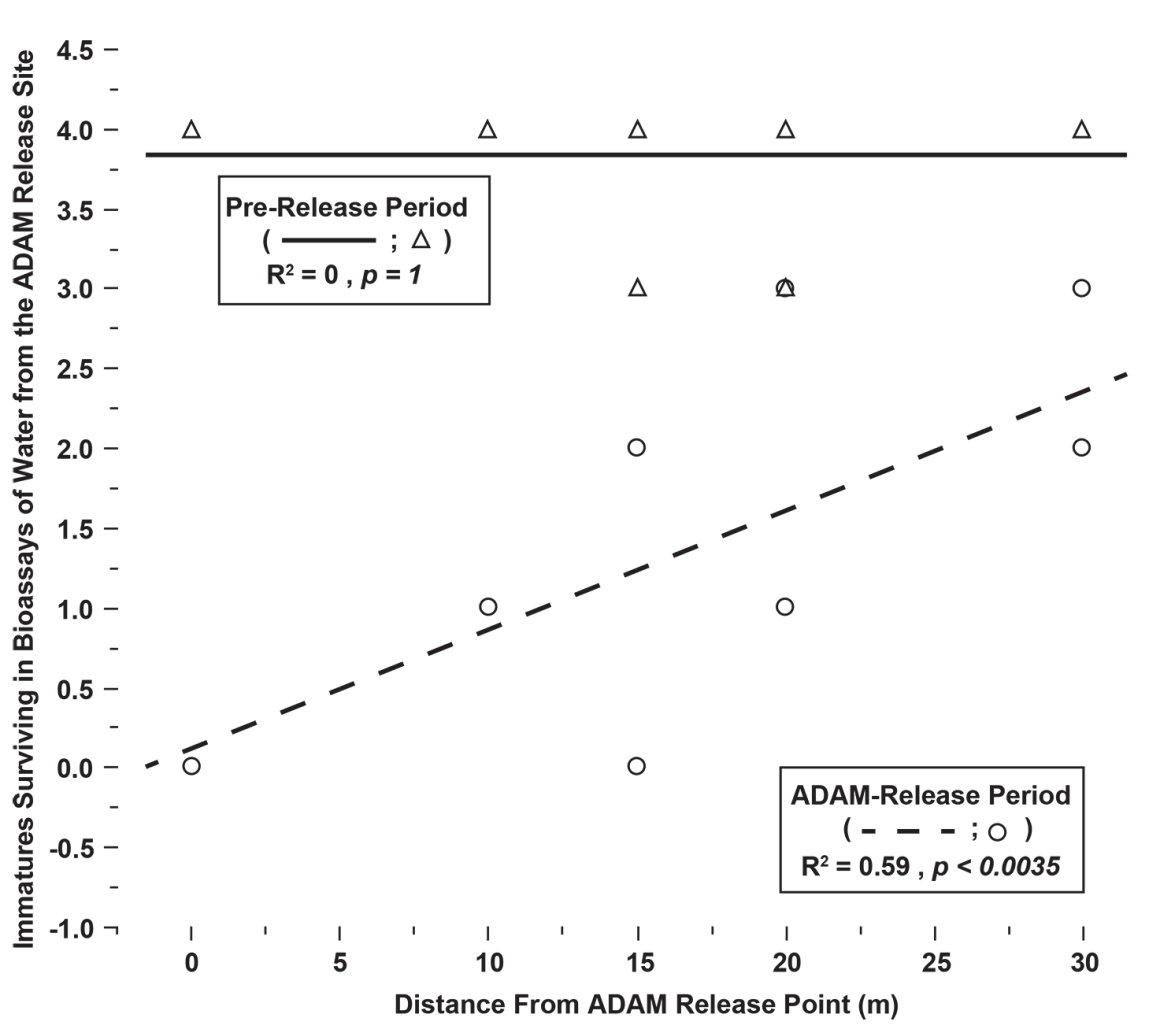
Immature survivorship in bioassays is correlated with distance from the introduction point in Field Trial 2, but only during the PPF-male introduction period. A bivariate fit of Survival versus distance is significant, but only during the two weeks of male introductions.

In the two week Pre-release period, prior to male introduction, good immature survival (89.6±19.4% survival, n = 24) resulted in bioassays of water sampled at both sites (Fig. 8), with no difference observed between the Treated and Untreated sites (p > 0.15, Wilcoxon). Subsequently, 6,300 and 5,100 PPF-treated males were introduced at the Treatment site in Weeks 3 and 4, respectively. During the two-week male introduction period, a significant difference was observed between the Untreated and Treated sites (Fig. 8). Bioassays of water sampled closest to the release point were completely lethal to immatures (0% survival). As shown in Fig. 9, a significant correlation was observed between bioassay lethality and distance from the release point during the introduction period, but not during the pre-introduction period. In contrast, high survival continued in assays of water from the Untreated site throughout all four weeks (Fig. 8). No females, *e*.*g*., indigenous population, were observed during the study.

## Discussion

The primary objective of this study was to assess the ADAM mosquito control strategy, which combines components of both autocidal and auto-dissemination approaches. Here, we have examined the ADAM approach against *Ae*. *albopictus*, a globally invasive species and important pathogen vector. The results demonstrate that, under the conditions tested here, *Ae*. *albopictus* males treated directly with PPF do not suffer a measurable decrease in longevity, relative to untreated males. In field cage trials, PPF dust was transferred from males to females and to larval breeding sites, at levels adequate to reduce or eliminate immature survival. Field trials show that the introduced males can quickly disseminate PPF, both in the absence and presence of female *Ae*. *albopictus*. Under the conditions tested, the PPF persists for at least six days after the males were dusted.

Bioassay results show that females in the field cage experiment become contaminated with PPF dust. Because the adult females were not treated with PPF, the contamination of females necessarily resulted from cross-contamination by males. An obvious opportunity for horizontal transfer between males and females is via coupling during mating and mating attempts. This type of transfer would be similar to that described in previous work with auto-dissemination stations [18]. Indirect transfer to females, via resting surfaces, *e*.*g*., water or ovisite sides (Fig. 3), is an additional potential route for female contamination with PPF.

Early season field trials demonstrate that male *Ae*. *albopictus* can directly deliver PPF to ovisites, in the absence of females, and at levels that are lethal to immatures. Male mosquitoes are understudied in general, and their behavior relative to breeding sites has not been well defined. Presumably, male *Ae*. *albopictus* may visit ovisites for hydration or as favorable, humid microhabitat refuges. Or perhaps the male behavior of visiting ovisites can be adaptive, by increasing the frequency of female encounters and mating opportunities. This represents an area for additional study.

An ability of males to directly deliver compounds to breeding sites can provide useful functionality, relative to auto-dissemination approaches that rely upon indigenous mosquitoes to communicate the active ingredient from the introduced station. By using laboratory-reared, male mosquitoes as vehicles, the ADAM approach can be deployed in areas that have relatively low indigenous mosquito densities. As an example, our results show that introduced males can intoxicate potential breeding sites, before the seasonal emergence of the indigenous population. This can allow anticipation of a seasonal increase, which can accelerate application, relative to an approach that is dependent upon the indigenous population. Direct treatment of laboratory-reared males allow for uniform application of the pesticide under controlled conditions. There is no need to deploy or maintain auto-dissemination stations.

The results show that, in addition to direct transmission of lethal compounds, males can cross-contaminate females, at dosages that are lethal to developing mosquitoes. The subsequent transmission to breeding sites by females is similar to that of traditional, station-based auto-dissemination approaches. Female transmission can be an important component, because ovipositing females can visit multiple larval breeding sites, treating each with a toxic dose. Additional downstream work, ideally with observations occurring in the field, will help to better define the transfer pathways and their relative importance.

The results support the continued development of the ADAM approach and open field trials in which PPF-treated *Ae*. *albopictus* males are released to directly contaminate *Ae*. *albopictus* females and breeding sites. While here we have tested the approach against *Ae*. *albopictus*, similar work can examine the utility of this approach against additional medically important species of mosquitoes, *e*.*g*., *Ae*. *aegypti*, *Culex pipiens*, and *Anopheles spp*. Because different species can share common breeding sites, *e*.*g*., it is common to find both *Ae*. *aegypti* and *Ae*. *albopictus* in the same larval habitats [36], an approach based on the introduction of PPF-treated *Ae*. *albopictus* males can affect populations of both *Ae*. *albopictus* and *Ae*. *aegypti*.

PPF-treated or not, an ADAM approach based on an exotic species is less likely to be adopted as a control tool. Therefore, adapting the ADAM approach to indigenous species can facilitate its use in a broader range of geographic areas. We note that the species used in an ADAM approach need not be mosquitoes or males necessarily. The key decision factors in species selection will include that the released insects (1) should not cause harm, *e*.*g*., bite or transmit pathogens or be an agricultural pest, (2) should deliver, directly or indirectly, the insecticide to the larval breeding sites of the targeted insect species, and (3) should be colonized and relatively easy to manage and rear.

Similar to other insecticidal approaches, the issue of effects to non-target organisms must be considered, *e*.*g*., potential for affecting larval competition. The amounts of PPF used via the ADAM approach are likely to be similar to that of station-based auto-dissemination strategies, and likely to be less than that of a human applied, broadcasting approaches. While the use of alternate active ingredients in the ADAM approach can be envisioned, the characteristics of pyriproxyfen make it an interesting candidate. These features include its high toxicity to immature mosquitoes, low toxicity to adult mosquitoes, a substantial amount of prior research and environmental assessment, and its classification as a low risk insecticide [29]. The non-target organism toxicity characteristics of PPF compare favorably with many other approved insecticides [37–40]. PPF effects are mainly at the pupal stage, when mosquitoes are not feeding and resource competition is less. This late-acting effect can be advantageous in mitigating density-dependent effects, which can offset the impact of earlier-acting compounds [41,42]. An additional feature of PPF that may be examined in future ADAM-related work are its effects on female fertility and male spermiogenesis [23,43–45]. Specifically, PPF contamination of females can reduce fertility of females and males, which can negatively impact the population in addition to the pupacidal effect of PPF [46].

We envision that an ADAM approach would be one component of an integrated vector management strategy. The strengths of the ADAM approach would be that of (1) ‘self-delivery,’ similar to other autocidal approaches, and (2) the ability of mosquitoes to find/treat cryptic breeding sites, similar to other auto-dissemination approaches. The small dosages delivered by the ADAM approach are less likely to affect large-volume pools, ponds, etc. But the latter are a strength of existing, traditional larviciding strategies. Adulticiding will continue to be needed for quick knock down of the adult mosquito population, but appropriate operational timing can allow for the integration of many adulticiding approaches with the ADAM approach, and alternation of different active ingredients can help to mitigate insecticidal resistance.

Similar to additional autocidal approaches, the *Ae*. *albopictus* ADAM approach will require large-scale production, *i*.*e*., ‘mass rearing,’ of mosquitoes for release. This type of mass rearing operation is developed already and in use with other autocidal approaches, including Sterile Insect Technique (SIT) [47–49]. Furthermore, the potential benefit of PPF treatment to ‘boosting’ autocidal approaches has been highlighted previously [50].

Here we have examined a new approach against mosquitoes, which combines components of both auto-dissemination and autocidal methods. Clearly, there is need for additional, larger field trials, conducted within different ecological contexts and culminating in community-randomized controlled trials. Relative to station-based auto-dissemination approaches, attractive features of the ADAM approach include (1) the ability to directly apply larvicidal compounds and thereby avoid complicating variation caused by mosquito self-treatment and variable environmental conditions; (2) an ability to regulate the size and location of treated-male introductions, expanding the utility to areas where mosquito populations are low, *e*.*g*., early in the season; and (3) avoiding the requirement of placing, maintaining and recovering dissemination stations.

The classification of PPF as a low risk compound, its relatively low environmental impacts [23,29], its residual activity (4 months in water) [25], and absence of resistance in mosquito populations [26] help to make PPF an attractive candidate for the ADAM approach. The susceptibility of multiple species of Aedes, Culex and Anopheles [51] and the fact that multiple mosquito species can share the same breeding sites [12] allow this approach to be extended to additional, medically important systems, with the potential to impact dengue, malaria, filariasis, chikungunya and additional mosquito-borne pathogens.

## Supporting Information

S1 Fig Model predictions are that the success of the auto-dissemination approach depends on mosquito activity. Therefore the model (Equation 1) predicts that an auto-dissemination approach that is reliant on indigenous mosquitoes will (1) be relatively ineffective in areas of low mosquito activity and (2) can become a victim of its own success. With fewer mosquitoes, fewer ovipositions (*O*) will occur. Assuming that the number of potential breeding sites (*H*) and insecticide potency (Ω) remain constant, fewer mosquitoes will result in fewer ovipositions/habitat and lower coverage of breeding sites (*C*_*h*_). This pattern is consistent despite the durability of the pesticide (*U*) [19].
(TIF)

S2 Fig Simple linear regressions of the adult female collections over time by site. Untreated Sites 1 and 2 are non-significant (*p* > 0.9) and significantly positive (*p* < 0.049), respectively. Site 3, which was treated with ADAM males, declined significantly (p < 0.0001) following treatment.
(TIF)
