# Supplementary figures and images for: Male Mosquitoes as Vehicles for Insecticide
Source: PLoS Negl Trop Dis. 2015 Jan 15;9(1):e0003406. doi: 10.1371/journal.pntd.0003406 (PMC4322094; doi:10.1371/journal.pntd.0003406)

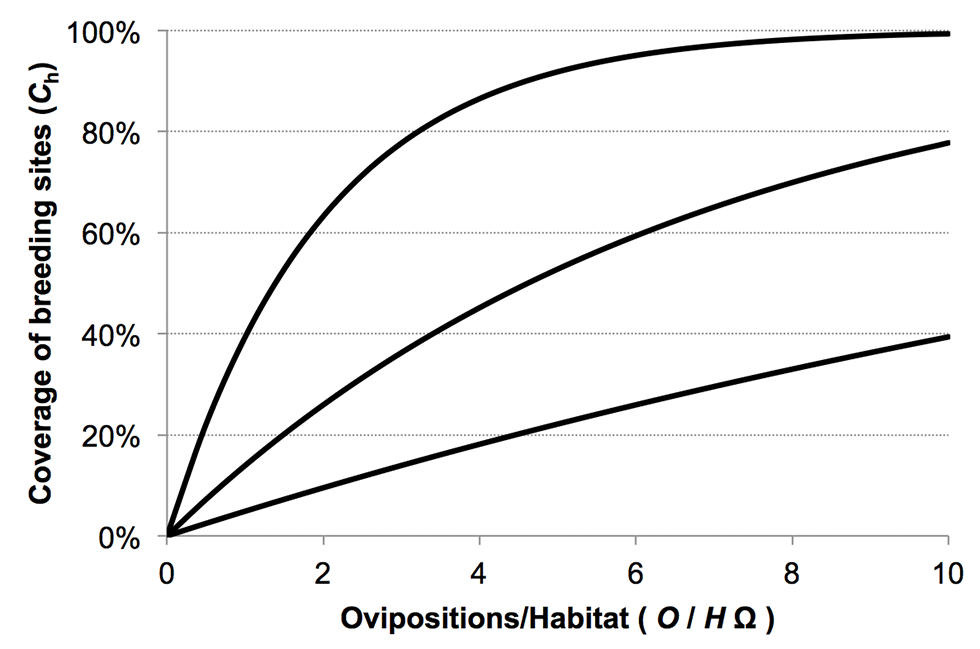

Supplement: S1 Fig — Therefore the model (Equation 1) predicts that an auto-dissemination approach that is reliant on indigenous mosquitoes will (1) be relatively ineffective in areas of low mosquito activity and (2) can become a victim of its own success. With fewer mosquitoes, fewer ovipositions (O) will occur. Assuming that the number of potential breeding sites (H) and insecticide potency (Ω) remain constant, fewer mosquitoes will result in fewer ovipositions/habitat and lower coverage of breeding sites (C h). This pattern is consistent despite the durability of the pesticide (U) [19]. (TIF) [file pntd.0003406.s001.tif]

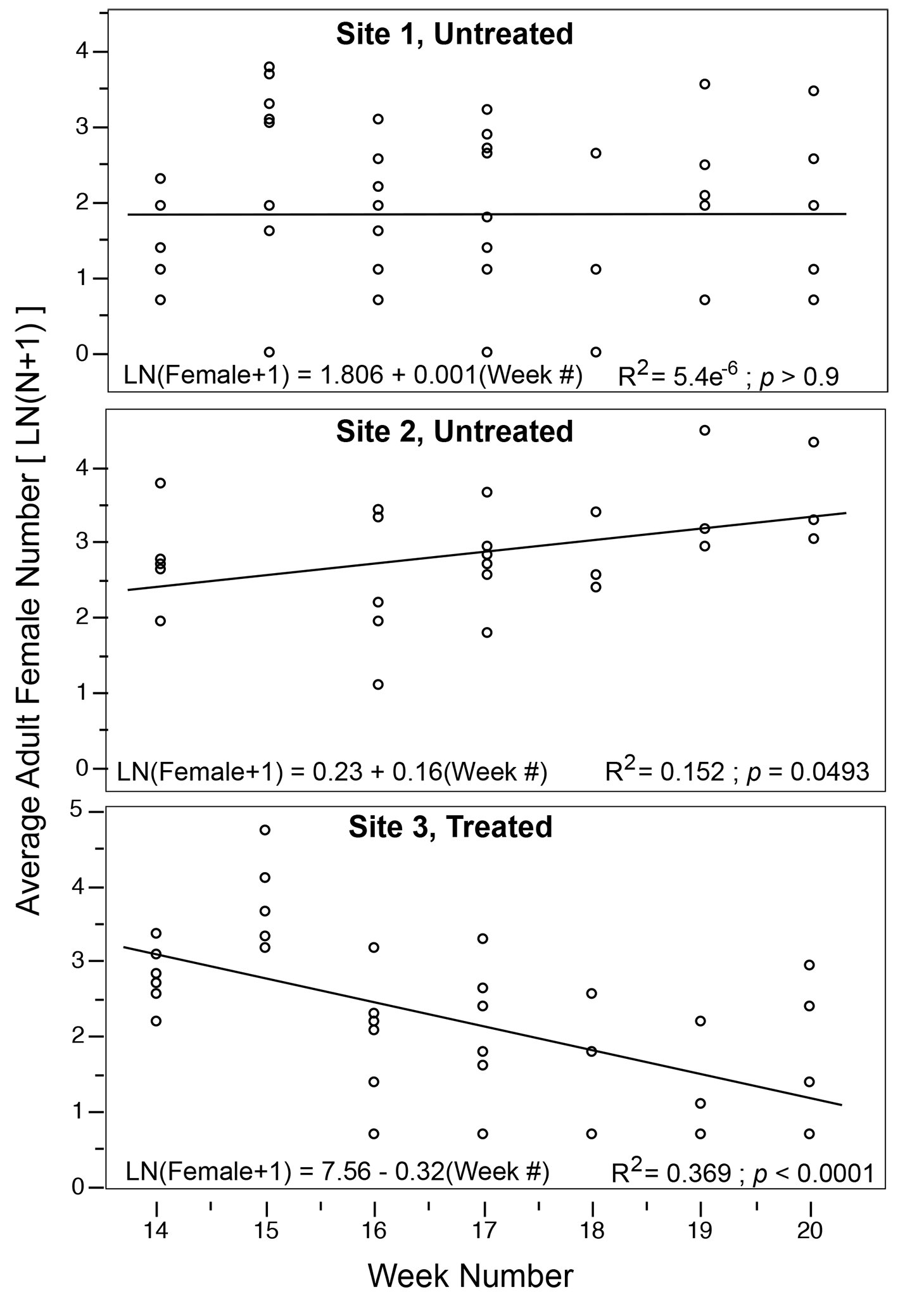

Supplement: S2 Fig — Untreated Sites 1 and 2 are non-significant (p > 0.9) and significantly positive (p < 0.049), respectively. Site 3, which was treated with ADAM males, declined significantly (p < 0.0001) following treatment. (TIF) [file pntd.0003406.s002.tif]
